# Exploring autonomous methods for deepfake detection: A detailed survey on techniques and evaluation

**DOI:** 10.1016/j.heliyon.2025.e42273

**Published:** 2025-01-25

**Authors:** Reshma Sunil, Parita Mer, Anjali Diwan, Rajesh Mahadeva, Anuj Sharma

**Affiliations:** a Department of CSE, Marwadi University, Rajkot, 360003, Gujarat, India; b Department of CSE, Manipal Institute of Technology, Manipal Academy of Higher Education, 576104, Manipal, India; c Jindal Global Business School, O. P. Jindal Global University, Haryana, India

**Keywords:** Deepfakes, CNN, Artifacts, Deep learning, Machine learning, Face swap, Facial reenactment, Synthetic media, GANs, Autoencoders, Digital media forensics

## Abstract

The fast progress of deepfake technology has caused a huge overlap between reality and deceit, leading to substantial worries over the authenticity of digital media content. Deepfakes, which involve the manipulation of image, audio and video to produce highly convincing yet completely fabricated content, present significant risks to media, politics, and personal well-being. To address this increasing problem, our comprehensive survey investigates the advancement along with evaluation of autonomous techniques for identifying and evaluating deepfake media. This paper provides an in-depth analysis of state-of-the-art techniques and tools for identifying deepfakes, encompassing image, video, and audio-based content. We explore the fundamental technologies, such as deep learning models, and evaluate their efficacy in differentiating real and manipulated media. In addition, we explore novel detection methods that utilize sophisticated machine learning, computer vision, and audio analysis techniques. The study we conducted included exclusively the most recent research conducted between 2018 and 2024, which represents the newest developments in the area. In an era where distinguishing fact from fiction is paramount, we aim to enhance the security and awareness of the digital ecosystem by advancing our understanding of autonomous detection and evaluation methods.

## 1 Introduction

The emergence of deepfake technology has initiated a novel era in which the integrity of digital media is progressively vulnerable to manipulation. The phrase “deepfake” refers to the application of artificial intelligence (AI) [1,2] and machine learning techniques to create or alter videos, images, or audio recordings in a way that appears genuine, although fabricated. The technology indicated above has attracted significant attention due to its vulnerability to misuse, including actions such as spreading false information, creating fake news items, and manipulating personal identities. Deepfakes, which utilize sophisticated machine learning algorithms and cutting-edge computer vision techniques, possess the capability to generate highly realistic multimedia content that has the potential to trick both human observers and automated verification systems [3]. As the advancement of this technology progresses, its ramifications for the credibility of media, the nature of political discourse, and the preservation of personal privacy grow progressively significant and concerning.

Recent years have witnessed an increase in the number of surveys and summaries about Deepfakes, along with the detection approaches discussed in academic literature. Jiaxin Ai et al. [4] introduced face deepfake and proposed a deep-learning-based approach called DeepReversion. It uses UNet to map the deepfake face to the original face, and experiments on public deepfake datasets show that the predicted face is highly consistent with the original face in terms of visual effects, PSNR, SSIM, and similarity. Anuwat Chaiwongyen et al. [5] look into how timbre and shimmer sound features can be used to tell the difference between real and fake speech. This difference is made up of eight audio components and four shimmer components. These features were used to test a method for finding deepfake speech using a dataset from the Audio Deep Synthesis Detection Challenge. Yang Hou et al. [6] proposed a statistical consistency attack (StatAttack) to minimize statistical differences in DeepFake detectors. It involves adding statistical-sensitive natural degradations to fake images, optimizing different degradations using a distribution-aware loss, and extending the attack to MStatAttack. The method has been tested on spatial-based and frequency-based detectors, demonstrating its effectiveness in both white-box and black-box settings. Abu Qais et al. [7] proposed a speech spoofing detection system using Convolutional Neural Networks to classify human speech and synthetic voices. The system uses 2D graphs to represent audio signals, reducing computation. The system can detect deepfake voices by converting audio into images of audio features and obtaining numeric values. Different approaches are used for individual and combined prediction.

Muxin Pu et al. [8] introduced metamorphic testing to evaluate the reliability of a deepfake detection model, MesoInception-4, and its impact on output. The model is used to detect makeup as anomalies, and the results are analyzed to identify potential gender biases in deep learning and AI systems. The study aims to explore whether the MesoInception-4 model produces unfair decisions due to robustness issues. Chang-Sung Sung et al. [9] presented an Audio-Visual Temporal Synchronization for Deepfake Detection framework for detecting deepfakes while maintaining detection capabilities for unseen ones. The framework evaluates the consistency between sound and faces in a video clip, using a spatiotemporal feature extraction network and a temporal classifier network. The model is trained on forged data to prevent overfitting and has been tested on unseen forgery categories. Bo Zou et al. [10] proposed a simple contrastive pertaining framework for DeepFake detection (DFCP) that finetunes after pretraining and requires only 5 percent labels. The framework learns high-frequency texture features and semantics information simultaneously, using a video-based frame sampling strategy to mitigate noise. Experimental results show high efficiency at frame-level, outperforming video-level methods. Hefei Ling et al [11] proposed a Local-Prediction framework that allows image-level labels to supervise local regions, introducing the Local-Diversity concept in Deepfake detection. They introduce the Local-Diversity Loss to enrich local features and limit each classification unit's receptive field. The method is evaluated on three benchmark datasets and shows significant performance for different CNN backbones. The proposed Local-Diversity Loss enriches binary classifier learned patterns, and visualization and ablation studies are provided for understanding the mechanism. Yun Huang et al. [12] introduced DF-VLAD, a VLAD-based aggregation module that allows numerous frames to be aggregated from the output layer to the feature layer. This module employs forgery detection to direct frame-level depth representation learning. The paper addressed a classification problem with fine-grained distinctions between fake and genuine faces. Existing face forgery techniques leave comparable spatial artifacts, whereas natural faces have more similar features. The paper proposes a model for forgery trace capture that combines self-attention and channel attention mechanisms, with note intentions guiding the network.

This survey paper differentiates itself from other recent works by providing a comprehensive and up to date review of deepfake detection technologies, focusing exclusively on advancements made between 2018 and 2024. Unlike many surveys, it covers a wide range of deepfake types, including images, videos, audio, textual, and real-time content, along with their generation and detection techniques. The paper integrates a detailed case study from 2023, highlighting a real-world financial fraud incident caused by deepfake technology, which underscores the immediate societal impact of such threats. It explores emerging detection methods like spatiotemporal analysis, GAN fingerprinting, and audiovisual consistency checks, offering insights into their strengths and limitations. The inclusion of structured evaluations, such as tables comparing datasets, tools, and performance metrics, makes it easier for readers to identify research gaps and opportunities. Beyond the technical aspects, the paper delves into ethical implications, societal challenges, and diverse applications of deepfake technology across industries, providing a holistic perspective. The use of visualizations, such as figures illustrating dataset distributions and detection approaches, further enhances the clarity and accessibility of the content. Overall, this paper stands out as a thorough and well-organized resource for understanding the latest trends and challenges in deepfake detection.

The succeeding portions of the paper are structured as follows. Section.

II of the document delineates the various classifications of deepfake technology. Section III of the paper encompasses the generation of deepfake technology. Deepfake detection techniques along with a comprehensive overview of the methodologies employed for detecting deepfakes is presented in Section IV. The table in Section V provides a comprehensive summary of forgery types and its detection techniques. Section VI presents a case study on deepfake. Section VII includes the applications of Deepfake Technology. Section VIII contains the ethical implications of Deepfake. Finally, we draw a conclusion and discuss potential avenues for future research in section IX.

## 2 Types of deepfake

**Fig. 1:**
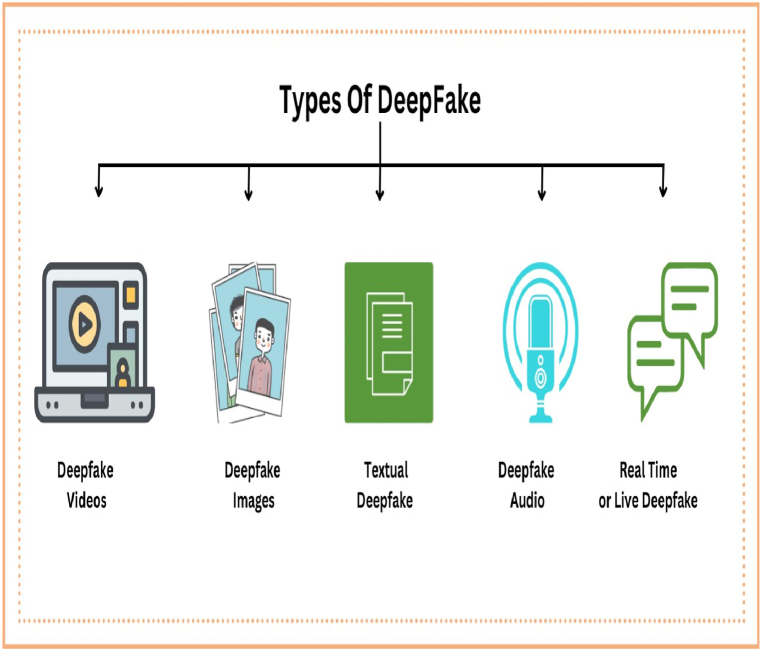
Types of deepfake deceits.

The word “deepfake” is coined by combining the concepts of “deep learning” and “fake” and it serves to denote both the nature and the methodology behind their creation [13]. Deepfakes refer to content alterations that are powered by artificial intelligence. These entities are produced in diverse media, encompassing textual, audio, video, images, and real-time streams that are shown in Fig. 1, which are further explained in the subsequent subsections. Table 1 shows the types of deepfake with its estimated usage percentage with the research focus.

**Table 1:** Types of Deepfakes and their Research Focus.

Deepfake Type	Estimated Percent- age	Why It's Used	Why Researched More	Examples	Research Focus
Video	50–60 %	High impact for manipulating reality. Can be humorous, satirical, or malicious.	High potential for misuse, complex to create realistically.	Celebrity deepfakes, political disinformation, creating fake news events.	GANs, deep learning architectures for video generation.
Image	30–40 %	Effective for creating fake news or social engineering scams.	Easier and faster to create than video deepfakes, significant impact on social media.	Altered photos of people or products, creating fake profiles.	GANs, autoencoders/VAEs for image manipulation.
Audio	5–10 %	Can be used to impersonate voices for scams or create fake interviews.	Technological advancements making audio deepfakes more realistic, potential for financial fraud.	Spoofing voice messages for financial gain, creating fake celebrity endorsements.	WaveGAN, audio deep learning techniques for speech synthesis.
Textual	1–5%	Can be used to generate fake reviews, news articles, or social media posts.	Emerging technology, easier to detect inconsistencies compared to visual/audio deepfakes.	Spam bots spreading misinformation, creating fake marketing content.	Natural Language Processing (NLP) techniques for text generation.
Real-Time	Less than 1 %	Emerging technology with potential for entertainment (live filters) or malicious use (impersonating someone in a video call).	Highly technical challenge, limited real-world applications yet.	Live manipulation of facial expressions in video calls, creating fake live events.	Real-time deep learning architectures for video manipulation (limited research).

### 2.1 Deepfake videos

Deepfake videos encompass a category of manipulated or fabricated videos wherein the facial features of an individual are substituted or modified within preexisting video content, creating the illusion that the person in question is engaging in actions or uttering statements that they have not actually performed. In order to generate deepfake videos, it is commonly necessary to train a deep learning model using a substantial dataset consisting of photos and videos featuring both the target individual (whose face will be substituted) and the source individual (whose face will be employed for the substitution) [14,15]. The model acquires the ability to understand and establish a correspondence between the facial characteristics of the subject individual and the facial appearance of the reference human, hence enabling the generation of authentic face exchanges.

After the completion of training, the deep learning model has the capability to generate novel video content through the sequential replacement of each frame's facial features of the target individual with those of the source individual. The outcome of this process yields a film whereby the visage of the primary subject is smoothly substituted for the initial countenance, replicating their facial expressions, physical gestures, and verbal communication. The rising popularity of deepfake videos can be attributed to their capacity for exploitation and manipulation.

### 2.2 Deepfake images

Deepfake images refer to images that have undergone modifications or construction through the utilization of deep learning methodologies, particularly Generative Adversarial Networks (GANs) [16,17]. The techniques encompass the process of training a model using a substantial dataset of authentic photos, followed by utilizing the trained model to generate novel images that possess a very lifelike appearance, although being wholly synthetic. Deepfake images give rise to substantial ethical considerations. These platforms have the potential to propagate misinformation, generate revenge pornography, or engage in character assassination by subjecting individuals to humiliating circumstances. The utilization of deepfake images has the potential to further amplify the problem of false information and disinformation. The growing recognition of deepfakes necessitates a heightened emphasis on the verification of visual content's authenticity as a prerequisite for belief or dissemination.

### 2.3 Textual deepfake

Textual deepfakes encompass the production of persuasive writing by leveraging artificial intelligence (AI) and natural language processing methodologies. The deepfakes in question are to AI systems that possess the ability to generate written content, encompassing articles, poems, stories, and other forms of textual compositions, which closely mimic the style and structure of text produced by humans. Textual deepfakes employ advanced language models to generate coherent and contextually appropriate textual content in response to a certain prompt or topic [18]. The models utilized in this context undergo training using extensive datasets in order to identify patterns in language and produce text that closely emulates human-authored content.

Textual deepfake systems possess the capability to produce a whole article or story that exhibits the semblance of human authorship, upon being provided with a headline or topic. These systems possess the ability to understand subtle subtleties in language, adhere to a coherent logical structure, and replicate the writing style of specific authors or literary genres [19]. The emergence of technological advancements in the field of textual deepfakes has elicited apprehension around the dissemination of misinformation, instances of plagiarism, and the potential for generating very persuasive counterfeit news items.

### 2.4 Deepfake audio

Deepfake audio, similar to deepfake images, pertains to audio that has been modified or synthesized through the utilization of deep learning methodologies. These methodologies encompass the process of training a model using an extensive dataset comprising authentic audio recordings [20]. Subsequently, the trained model is utilized to produce novel audio outputs that mimic the vocal characteristics and speech patterns of a particular individual. The production of deepfake audio generally involves the utilization of a deep learning model, such as a Recurrent Neural Network (RNN) [21] or WaveNet [22], which is trained on a dataset comprising numerous speech recordings of the specific subject being targeted. This enables the model to produce novel audio that exhibits a strong resemblance to the voice of the target individual. Detecting deepfake audio poses a significant challenge because of the highly persuasive outcomes produced by these techniques.

### 2.5 Real-time or live deepfake

The term “real-time” or “live” deepfake pertains to the ability to generate deepfake information instantaneously or in real-time, rather than modifying pre-existing media [23]. This emerging technology combines computer vision, machine learning, and graphics processing capacity to change and alter both audio and visual content in real-time. Real-time deepfakes find utility in various domains such as live video manipulation, interactive entertainment, virtual reality, and augmented reality experiences. These technologies enable real-time face-swapping and manipulation of facial emotions in video chats and live broadcasts [24].

The emergence of real-time deepfakes has given rise to significant concerns around privacy, consent, and potential misuse. In the absence of adequate regulation and ethical considerations, this technology has the potential to be utilized for purposes such as impersonation, harassment, and misleading tactics. There is a significant number of developers who are now involved in the advancement of real-time deepfake technologies as well as the creation of detection techniques aimed at identifying and mitigating the impact of real-time deepfakes. The primary goal is to attain an ideal balance between the advancement of technology and the assurance of its ethical and responsible implementation.

## 3 Deepfake Generation Process and its techniques

**Fig. 2:**
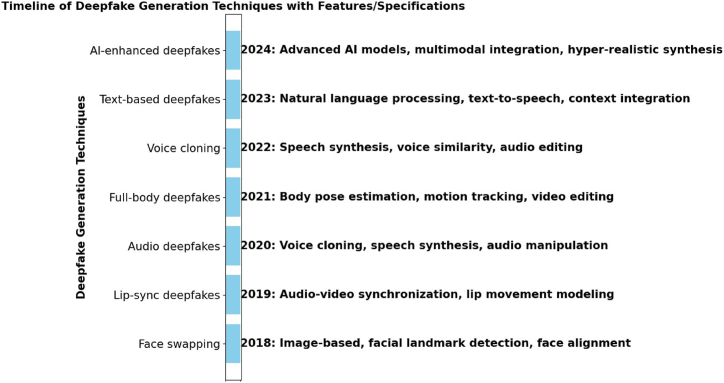
Timeline of deepfake generation techniques with features/specifications.

The process of generating deepfakes involves the utilization of artificial intelligence (AI) techniques, specifically deep learning algorithms. Deepfakes, a term derived from “deep learning” and “fake”,denote the production of synthetic or manipulated multimedia content that is often remarkably persuasive, encompassing videos, images, or audio recordings. Fig. 2 shows the timeline ranging from 2018 to 2024 of deepfake generation techniques with features and specifications representing the growth and trend change.

### 3.1 Deepfake Generation Process

**Fig. 3:**
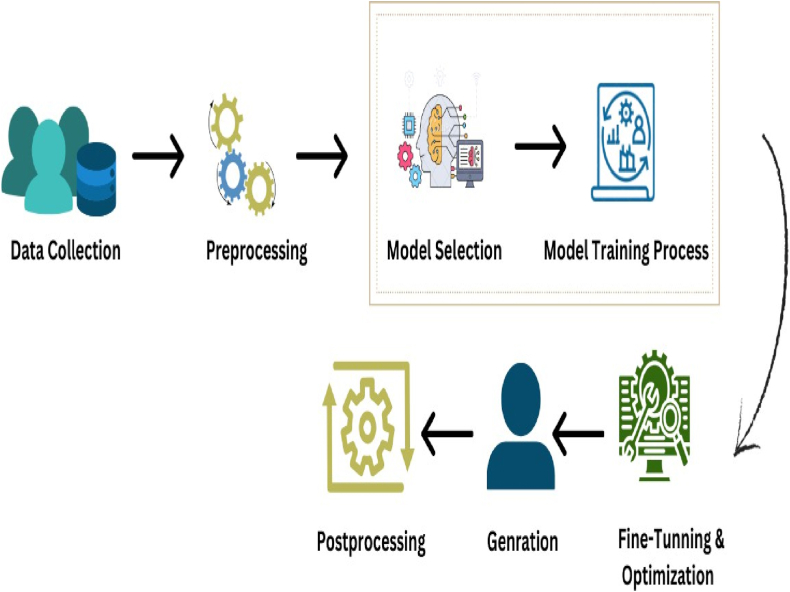
Deepfake generation process.

To generate deepfakes, a combination of deep learning algorithms and neural networks, specifically generative models, is employed as shown in Fig. 3. The following is a step-by-step summary of the typical process involved in generating deepfakes.

• **Data Collection:** Deepfake generation begins with the acquisition of a substantial quantity of training data for neural networks. This information may include photographs, videos, or audio recordings of the person(s) whose likeness or voice the creator wishes to impersonate. The quality and quantity of the data have a significant impact on the quality of the deepfake that is generated.
• **Preprocessing:** The collected data is preprocessed to guarantee consistency and compatibility. This may involve resizing, cropping, or aligning images to ensure that features are centered and proportionally sized. Noise reduction and audio alignment can be used to enhance the quality of audio recordings.
• **Model Selection:** The creator of deepfake selects a suitable generative model. Variational Autoencoders (VAEs) [25,26], Generative Adversarial Networks (GANs), and specialized models designed for specific tasks such as face-swapping and voice synthesis are popular options.
• **Training the Model:** Training the model includes major two approaches Gan-based and Autoencoders-Based approach [27]. In GANs, the generator and discriminator are the two essential components. The Generator learns to generate content (e.g., images, audio) whose characteristics resemble those of the target. It transforms random noise or another input (such as an initial face image) into a deepfake. The Discriminator is trained to differentiate between genuine and artificial content. It assesses the quality and authenticity of the generated deepfake. In Autoencoders Based Approach, Autoencoders, such as VAEs, learn to encode and decode input data. Deepfakes are created by encoding actual data (e.g., faces) and then modifying the encoded representations.
• **Fine-Tunning and Optimization:** The training process is frequently iterative and may entail fine-tuning the model and optimizing hyperparameters in order to enhance the quality and realism of the deepfake.
• **Generation:** Once the model has been trained, it can be used to generate new deepfake content by receiving the necessary input data or instructions. In a face-swapping scenario, for instance, the model might superimpose the target's visage onto the body of another person in a video.
• **Post-Processing:** Deepfake creators may perform post-processing to improve the authenticity and quality of the generated content but it is an optional step. This may involve noise reduction, color correction, or compositing to make the deepfake appear more realistic.

### 3.2 Deepfake generation techniques

Deepfake generating approaches utilize a range of artificial intelligence (AI) and machine learning (ML) methodologies to modify and synthesize audio-visual information [28]. Table 2 depicts the usage percentage of various deepfake generation techniques that are commonly used. Here are some of the key generation techniques commonly employed in creating deepfakes:

• **Autoencoders:** Autoencoders are a type of neural network that is commonly employed in the field of unsupervised learning. The system comprises of an encoder and a decoder, wherein the encoder compresses the input data (such as an image or audio) into a compressed form, and the decoder aims to recreate the initial data. Autoencoders have the capability to undergo training using a dataset consisting of authentic images or audio in order to effectively capture and represent patterns and features. Subsequently, individuals possess the capability to produce novel information that bears resemblance to the original training data, although frequently modified or manipulated, hence giving rise to deepfakes.
• **Variational Autoencoders (VAEs):** VAEs are a specific kind of autoencoders that introduce stochasticity into the encoding process. This characteristic renders them very suitable for producing a wide range of deepfake content through the sampling of the acquired latent space, employed in the context of deepfakes to incorporate a degree of unpredictability and diversity into the synthesized information. By employing the technique of sampling from the latent space, it is able to generate diverse variations of deepfake content, hence expanding the range of potential outcomes.
• **Generative Adversarial Networks (GANs):** GANs are composed of two fundamental components: a generator network and a discriminator network. The generator is designed to produce synthetic data, such as images or audio, with the objective of deceiving the discriminator into perceiving it as authentic. By means of an adversarial training procedure, GANs have the capability to generate progressively more persuasive deepfake content. GANs have gained significant prominence in the realm of deepfake creation, particularly in the generation of synthetic images and videos. The generator algorithm acquires the ability to generate information that progressively becomes more challenging for the discriminator algorithm to differentiate from authentic data, leading to the production of very persuasive deepfakes. The use of a competitive training procedure serves to augment the authenticity and fidelity of the generated content.
• **CycleGANs:** CycleGANs [29] are a specific variant of Generative Adversarial Networks (GANs) that are employed for the purpose of imageto-image translation problems, wherein the availability of paired training data is not required. The objective is to perform picture mapping between different domains while ensuring the preservation of fundamental properties. These technologies find application in artistic style transfers and the creation of image-based deepfakes. The ability to modify the artistic style or domain of images while maintaining their content enables the exploration of creative alterations.
• **WaveGAN and Parallel WaveGAN:** WaveGAN is one of the first GAN architectures to be developed for the generation of unprocessed audio waveforms. It was initially developed for the purpose of generating sound effects and audio synthesis, but it can be modified to perform voice synthesis and audio deepfakes. It operates directly on 1D raw audio waveforms, despite adhering to a structure that is similar to that of traditional GANs. The generator generates simulated waveforms, which are then assessed by the discriminator in comparison to genuine waveforms. It could potentially implemented to generate audio samples that are both false and realistic, such as voice deepfakes.

**Table 2:** Usage percentage of deepfake generation techniques.

**Technique**	**Usage Percentage (%)**	**Explanation**
**Generative Adversarial Networks (GANs) & Conditional GANs (cGANs)**	60–80 %	Research heavily focuses on GAN-based deepfakes due to their effectiveness in producing high-quality, realistic outputs.
**Supportive Techniques**		
- Autoencoders/VAEs	10–20 %	VAEs help with data representation for generating diverse variations.
- *CycleGAN*	10–20 %	CycleGAN aids in image-to-image translation and facial feature manipulation without paired examples.
- *WaveGAN*	10–20 %	WaveGAN specializes in audio data generation and manipulation.
**Foundational Techniques**		
- DNNs	5–10 %(combined)	These broad deep learning techniques form the foundation for deepfake architectures.
- RNNs	5–10 %(combined)	RNNs are used for sequential data, relevant for video and audio deepfakes.
- Transfer Learning	5–10 % (combined)	Transfer learning adapts pre-trained models for specific tasks, optimizing resources and time.
- Style Transfer	5–10 % (combined)	Style transfer techniques are used for artistic transformations and specific effects in deepfakes.

Parallel WaveGAN is intended to function as a high-quality, rapid vocoder that transforms mel-spectrograms into unprocessed waveforms. It was created to overcome the pace and quality constraints of conventional waveform generation models, such as WaveNet. Parallel WaveGAN synthesizes audio from mel-spectrograms by employing a GAN-based structure. It is highly efficient in comparison to autoregressive models due to the fact that it generates waveforms in parallel. It is notably advantageous for the creation of high-quality audio deepfakes, particularly when utilized in conjunction with text-to-speech (TTS) systems. It is well-suited for the production of real-time or large-scale audio deepfakes due to its capacity to rapidly generate realistic waveforms from spectrograms.

• **Deep Neural Networks (DNNs):** DNNs [30] are a type of neural network characterized by the presence of numerous hidden layers. Complex patterns in data can be captured by them. DNNs have been employed in the creation of text-based deepfakes, which are capable of generating highly authentic textual content that closely emulates the writing style of a particular individual.
• **Recurrent Neural Networks (RNNs):** RNNs are specifically engineered to handle sequential input, enabling them to effectively capture temporal dependencies within the data. RNNs play a crucial role in the generation of text-based deepfakes, such as fake news articles and chatbot interactions. RNNs receive extensive training on enormous text corpora, which can range from books and articles to social media posts. This training enables them to comprehend the patterns, relationships, and context of the language they encounter.
• **Conditional GANs:** Conditional Generative Adversarial Networks (GANs)
  [31]incorporate supplementary input data, such as an image or textual description, in order to condition and influence the generation process. Conditional GANs are commonly employed in many applications, such as face-swapping, where the input image serves as a guiding factor during the generation process. This ensures that the generated content is in alignment with the characteristics of the input image.
• **Transfer Learning:** Transfer learning is a technique that entails the refinement of pre-existing models by leveraging extensive datasets for the purpose of addressing specific problems [32]. Pre-existing models like Transformers [33], have the potential to undergo fine-tuning in order to facilitate the creation of text-based deepfakes. This process capitalizes on the models' inherent language comprehension ability to produce content that is contextually intricate.
• **Style Transfer Networks:** The primary objective of these networks is to facilitate the transfer of artistic style from one image to another [34]. Style transfer networks have the capability to combine the artistic style of renowned painters with authentic pictures, resulting in visually striking yet fabricated content.

## 4 Deepfake detection techniques

Deepfake detection refers to the process of recognizing manipulated or artifi-cially generated media content, such as videos or images, which have been generated through the utilization of deep learning methodologies [35]. The detection of deepfakes plays a pivotal role in upholding trust in media and mitigating the dissemination of inaccurate or deceptive content.

Deepfake detection involves collecting and preparing a dataset of real and potentially fake media content, ensuring format consistency, and extracting relevant features such as facial landmarks, audio spectrograms, and temporal data [36,37]. The dataset is then divided into training, validation, and test sets for evaluation and training purposes. Then, feature engineering is conducted to differentiate between authentic and deepfake content. An appropriate machine learning or deep learning model, such as Convolutional Neural Networks (CNNs) [38], Recurrent Neural Networks (RNNs), or hybrid models, is selected. Using the training dataset, the model is trained to distinguish between authentic and deepfake media by adjusting internal parameters. An in depth analysis of commonly used deepfake detection methods is shown in Table 3. Using the validation dataset, the efficacy of the model is evaluated, with hyperparameters such as learning rates and network architecture optimized. The efficacy of the model is evaluated using metrics such as precision, recall, and F1 score [39]. Post-processing techniques are utilized to refine the model's predictions, and a confidence score threshold is established to classify content as genuine or deepfake. In a real-world setting, such as a content-sharing platform or media

**Table 3:** In-depth analysis of Common Deepfake Detection Techniques.

Detection Technique	Strengths	Limitations	Potential Future Development
**Convolutional Neural** **Networks (CNNs)**	- Strong at de- tecting pixel-level anomalies. - High accuracy with large datasets.	- Requires extensive labeled data. - Computationally expensive.	- Development of lightweight CNNs for faster processing. - Improved generalization using transfer learning.
**Recurrent Neural Net-** **works (RNNs)/LSTMs**	- Effective for analyzing temporal inconsistencies in videos. - Good for time- series data.	- Requires large sequential datasets. - Less effective with short video clips.	- Integration with attention mechanisms for longer sequences. - Better handling of short, real-time videos.
**Optical Flow Analysis**	- Detects motion inconsistencies between frames.	- Limited to video deepfakes. - Sensitive to low- quality video data.	- Integration with deep learning for real-time detection. - Enhanced robustness to lower quality videos.
**Facial Behavior Analysis**	- Detects unnat- ural facial movements and behaviors. - Can spot anomalies in eye movement and blinking.	- Less effective with high-quality deepfakes. - Limited scope beyond facial detection.	- Broader scope for emotional analysis and multi-body behavior detection.
**Audio-Visual Inconsis-** **tency Detection**	- Identifies mismatches between speech and lip movement. - Useful for detecting video-based deepfakes.	- Requires high- quality synchronization of audio and video.	- Improved multi- modal fusion to handle more complex mismatches across formats.
**GAN Fingerprint Detec-** **tion**	- Identifies noise patterns or finger- prints left by GAN generation.	- GANs evolve, making this method less reliable over time.	- Adaptive GAN fingerprint detection to keep pace with newer GAN architectures. - Hybrid models combining GAN fingerprints with other techniques.
**Biometric-Based Detec-** **tion**	- Leverages heart rate or pulse through skin analysis.	- Ineffective in low-light settings or on non-facial deepfakes.	- Incorporation of additional biometric signals like thermal imaging or micro-expressions.
**Adversarial Training**	- Can improve against adversarial deepfake attacks.	- Needs frequent updates and is computationally expensive.	- Efficient ad- versarial training methods to handle real-time use cases.

verification system, the trained deepfake detection model is deployed. Continuous monitoring and updates are required to acclimate to the evolution of deepfake techniques and enhance precision. Also encouraged are user education and awareness about deepfakes and the significance of critical thinking. Consideration is given to human oversight for highly sensitive or critical applications. Deepfake detection is an ongoing challenge, and the accuracy of the detection system is dependent on the quality of the training data, the selection of the model, and the capacity to adapt to new deepfake techniques as they emerge. Fig. 4 represents the classification of deepfake detection techniques that are commonly employed in identifying forgeries in the digital media. Also, Fig. 5 shows the importance rating of different features in deepfake detection that plays a crucial role for the detection. The identification of deepfakes is a multifaceted approach that integrates several tools and techniques which are further briefed in the subsequent subsections:

**Fig. 4:**
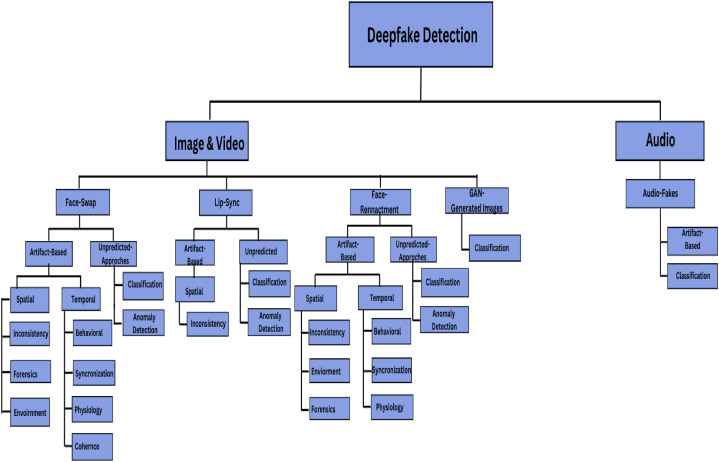
Classification of deepfake and its Detection.

**Fig. 5:**
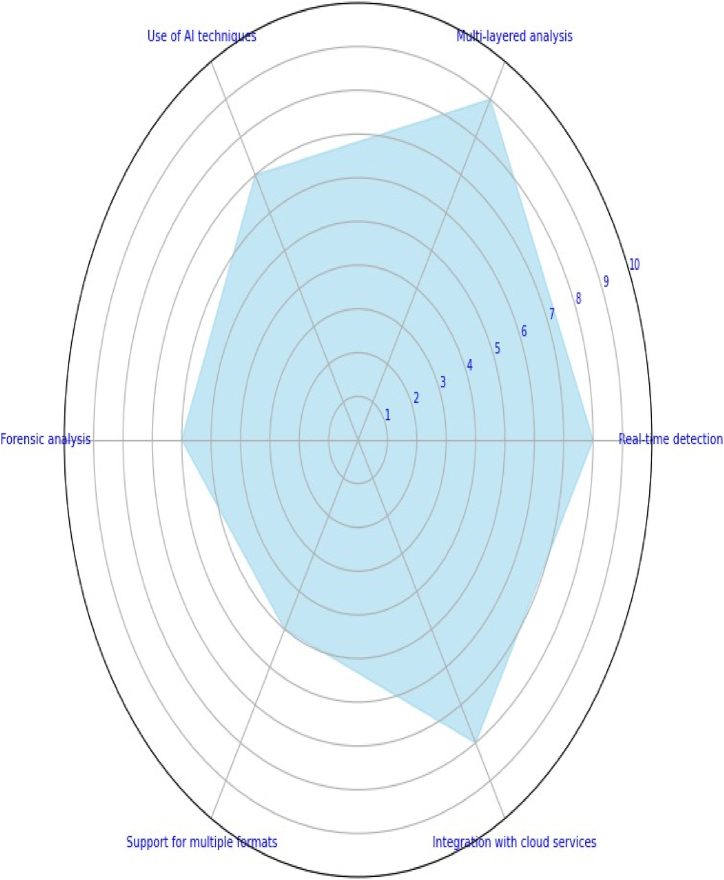
Importance of different features in deepfake detection.

### 4.1 Face and body analysis

Face and body analysis play a crucial role in the detection of deepfakes, which are videos or images created using artificial intelligence techniques that have been altered or fabricated. Face and body analysis can help identify anomalies and inconsistencies that may indicate the presence of a deepfake. Here are some essential facial and body analysis techniques and considerations for deepfake detection:

• **Facial Landmark Detection:** This technique identifies and tracks specific points on a person's face, such as the eyes, nose, mouth, and other facial features [40]. Deepfake detectors analyze the alignment and mobility of these landmarks over time using this information. These landmarks may not move organically or consistently in deepfake videos, indicating that the video has been manipulated.
• **Blink Analysis:** Blink analysis focuses on identifying blinking patterns in videos that are not natural. Deepfake detectors investigate how often and when a person blinks in a video. Blink patterns that exhibit anomalies or inconsistencies may indicate that a video is a deepfake.
• **Lip Synchronization Detection:** Lip synchronization analysis determines whether the audio and facial movements in a video are in sync. In deepfake videos, the vocal movements may not precisely correspond to the spoken words, which can be an indication of manipulation [41]. Deepfake detectors examine this synchronization in order to identify potential inconsistencies.

### 4.2 Image and video analysis

Techniques for image and video analysis are essential for detecting deepfakes, which involve the manipulation of visual and audio content. Deepfake detection employs a variety of techniques to analyze media and identify inconsistencies or anomalies that may indicate the presence of manipulated content. Here are some essential image and video analysis techniques and considerations for deepfake detection:

• **Inconsistencies in Resolution:** Deepfake images or videos might exhibit inconsistencies in resolution, sharpness, or noise levels. Using image processing techniques, these inconsistencies can be identified. Deepfake detectors may, for instance, search for abrupt changes in image quality within a video.
• **Temporal Analysis:** Temporal analysis is the study of the temporal consistency of facial expressions and movements throughout the duration of a video. The training of deep learning models to recognize patterns and variations in the behavior of a person's face over time. Inconsistencies or movements that are not natural can be indicators of a deepfake.
• **Metadata Examination:** Metadata [42] contain information regarding the creation, location, and modification history of an image or video. Deepfake detectors may examine this metadata to determine the authenticity of the content. Suspicious metadata, such as inconsistencies or outof-the-ordinary modification timestamps, may indicate manipulation.

### 4.3 Deep learning models

Deep learning models have proved effective for detecting deepfakes, as they are able to learn and recognize subtle patterns and inconsistencies in multimedia content. The following are common deep-learning models used for deepfake detection:

• **Convolutional Neural Networks (CNNs):** CNNs are models of deep learning typically used for image and video analysis. They are trained to recognize anomalies and patterns in images and videos. CNNs can be used to identify artifacts and irregularities left behind by generative models during deepfake detection.
• **Recurrent Neural Networks (RNNs):** RNNs are used to evaluate the temporal consistency of video frames. The ability of these networks to identify inconsistencies in facial expressions and movements across frames makes them useful for video-based deepfake detection.
• **Siamese Networks:** One-shot learning tasks employ Siamese networks [38]. In deepfake detection, it is possible to determine whether or not two images or video frames originate from the same source. This is helpful for identifying content that has been manipulated by comparing similarities between frames.

### 4.4 Generative model analysis

Generative models, particularly Generative Adversarial Networks (GANs), have played a major role in the creation of deepfake content. However, they can also be used to detect deepfakes, albeit in a different manner. Here is how generative models can be utilized to detect deepfakes:

• **Model Artifacts:** Deepfake generation models, such as Generative Adversarial Networks (GANs), frequently introduce particular artifacts into the generated content. These artifacts are unusual patterns or distortions that do not exist in actual images or videos. The detection of these artifacts may be indicative of a deepfake
• **Detection of GAN Noise:** GANs, which are frequently used to generate deepfake images, incorporate noise patterns into deepfake images. Analyzing these noise patterns can be a useful method for detecting GAN generated content.

### 4.5 Audio analysis

Audio analysis is an essential component of deepfake detection, particularly when manipulated audio is paired with fake visual content. Detecting inconsistencies in audio can aid in the identification of deepfakes that would otherwise be convincing. Here are some techniques and considerations for using audio analysis in the detection of deepfakes:

• **Source Verification:** Deepfake audio can be identified by analyzing the speaker's voice characteristics, such as pitch, tone, rhythm, and speech patterns. Inconsistencies between video and audio content, such as a voice that does not match the vocal movements, can be indicative of a deepfake.
• **Audio-Visual Synchronization:** This method ensures that the audio and video components of a video are perfectly synchronized. In deepfake videos, the audio may be out of sync with the vocal movements and facial expressions, indicating manipulation.

### 4.6 Data forensics and human expertise

Data forensics is the systematic examination of digital media for evidence of tampering, manipulation, and inconsistencies. In the context of deepfake detection, data forensics techniques are necessary for identifying traces of manipulation and validating the authenticity of multimedia content. Human expertise is a crucial aspect of deepfake detection because humans can recognize subtle nuances and contextual inconsistencies that automated algorithms might overlook.

• **Source Verification:** Digital forensics techniques are utilized by investigators to trace the source of an image or video. This involves analyzing digital traces, such as compression artifacts, metadata, and editing traces, to determine the content's origin and authenticity.
• **Human Verification:** In certain instances, human experts may manually examine content to identify inconsistencies or anomalies that automated tools may overlook. Especially in complex cases, human expertise is indispensable for verifying the veracity of suspect media content.

## 5 A side-by-side evaluation and a brief summary of interconnected research work

This section shows the various research works that we have surveyed in terms of tools, datasets, techniques, methodologies and performance metrics that are used for the purpose of deepfake generation and detection. Table 4, Table 5, Table 6 provides a comprehensive view of datasets, evaluation metrics and tools used in deepfake detection research, aiding in understanding the various approaches and resources available in the field. We have only collected data that are recent from a timeline of 2018–2024. The data included in this review were collected by synthesizing results from peer-reviewed research papers, academic studies, and large publicly available datasets. The selection criteria were based on the relevance to the topic of deepfake detection, the credibility of the sources, and the availability of detailed performance metrics.

**Table 4:** Datasets for deepfake detection.

**Dataset Name**	**Source/Provider**	**Number of Videos/Images**	**Annotations Available**	**Description and Usage**	**Unique Features**
FaceForensics++	Technical University of Munich	1000+ videos	Yes	Large-scale dataset with various manipulation methods.	Includes Deep-fakes, Face2Face, FaceSwap, and NeuralTextures.
DFDC (Deepfake Detection Challenge)	Facebook	5000+ videos	Yes	Diverse dataset for deepfake detection challenge	High diversity in subjects and environments.
Celeb-DF	University of Albany	590 videos	Yes	Celebrities' videos with real-istic deepfakes.	High-quality deepfakes with minimized artifacts.
UADFV (Utrecht Audio-Visual Deepfake Video)	Utrecht University	98 videos	Yes	Dataset with audio-visual deepfakes.	Focuses on audio and visual inconsistencies.
Google Deepfake Detection	Google/Jigsaw	363 real, 3068 fake videos	Yes	Dataset created to advance deepfake detection research.	Contains manipulated videos with various techniques.
DeepFake-TIMIT	University of Alberta	620 videos	Yes	Deepfake videos generated using the VidTIMIT dataset.	Different quality levels: low and high resolution.
DeeperForensics-1.0	SenseTime	10,000 videos	Yes	Large-scale dataset with controlled environment deepfakes.	Various perturbations and manipulations applied.
DF-VIVID	Indiana University	2000 videos	Yes	Deepfake videos with diverse subjects and settings.	Videos created with more recent deepfake generation techniques.
DFDC-preview	Facebook	1131 videos	Yes	Preview of the larger DFDC dataset.	Early access dataset for preliminary research.
WildDeepfake	Nanyang Technological University	7314 videos	Yes	Deepfake videos collected from the internet.	Reflects real-world deepfake scenarios with diverse sources.

**Table 5:** Evaluation metrics commonly used for deepfake detection.

Metric	Description	Formula	Use Cases
Accuracy	Measures the overall correctness of the model.	$\frac{T\;P+T\;N}{T\;P+F\;P+T\;N+F\;N}$	General performance assessment.
Precision	Measures the accuracy of positive predictions.	$\frac{T\;P}{T\;P+F\;P}$	Importance when false positives are costly.
Recall (Sensitivity)	Measures the ability to find all relevant instances.	$\frac{T\;P}{T\;P+F\;N}$	Importance when false negatives are costly.
F1-Score	Harmonic mean of Precision and Recall.	$2\times \frac{\text{Precision}\times \text{Recall}}{\text{Precision}+\text{Recall}}$	Balances precision and recall, useful for imbalanced classes.
Specificity	Measures the ability to identify only negative instances.	$\frac{T\;N}{T\;N+F\;P}$	Importance in detecting negative instances accurately.
AUC-ROC	Area under the Receiver Operating Characteristic curve.	N/A	Evaluates the trade-off between true positive rate and false positive rate.
Log Loss	Measures the performance of a classification model.	$\sum_{i=1}^{N}-\frac{1}{N}\;\left[yi\;\log\left(pi\right)+\left(1\;-yi\right)\;\log\left(1-pi\right)\right]$	Penalizes false classifications, sensitive to probabilistic predictions.
Confusion Matrix	Summarizes the performance of a classification algorithm.	N/A	Detailed analysis of classification performance across all classes.
True Positive Rate (TPR)	Measures the proportion of actual positives correctly identified.	$\frac{T\;P}{T\;P+F\;N}$	Similar to recall, often used interchangeably.
False Positive Rate (FPR)	Measures the proportion of actual negatives incorrectly identified.	$\frac{F\;P}{F\;P+T\;N}$	Important for understanding false alarm rate.

**Table 6:** Autonomous systems and tools commonly used for deepfake detection.

System/Tool	Developer/Source	Key Features	Performance
Deepware Scanner : **Open** **source**	Deepware	Real-time deepfake detection, user friendly interface, supports multiple video formats.	High accuracy on various deepfake datasets.
Sensity AI: **Commercial**	Sensity (formerly Deep trace)	Comprehensive deepfake detection, video and image analysis, extensive database of known deepfakes.	Industry-grade performance, low false positive rate.
XceptionNet: **Open source**	RISELab, UC Berkeley	CNN-based approach, high precision and recall, trained on large datasets.	State-of-the-art performance on FaceForensics++ dataset.
DeepFaceLab : **Open** **source**	Various Contributors	Deepfake creation and detection, customizable, extensive documentation.	Effective detection on self-created and external deepfakes.
VideoAuth: **Commercial**	Amber Video	Multi-layered detection, combines forensic and AI techniques, realtime processing.	High accuracy, robust against various manipulation techniques.
FaceNet2ExpNet : **Open** **source**	University of Tartu	Focuses on expression transfer detection, uses facial recognition techniques.	High performance in expression manipulation detection.
ForensicTransfer : **Open** **source**	Nanyang Technological University	Transfer learning-based approach, detects low-quality deepfakes, interpretable model.	Competitive performance on challenging datasets.
TwoStreamNet : **Open** **source**	Technical University of Munich	Two-stream network (RGB and optical flow), robust against compression artifacts.	High accuracy and robustness on FaceForensics++.
DeepFake-ometer: **Open source**	University of Campinas	Measures the probability of video being a deepfake, easy-to-use interface.	High detection rate, low computational cost.
MesoNet : **Open** **source**	Universit'e Côte d’Azur	Lightweight CNN, specifically designed for deepfake detection, fast and efficient.	Good performance with low computational requirements.
DeepFakeShield : **Commercial**	Microsoft Azure	Cloud-based deepfake detection, integrates with Azure services, realtime analysis.	Scalable and robust performance in cloud environments.
DefakeHop++ : **Open** **source**	University of Southern California	Lightweight and efficient, uses Successive Subspace Learning (SSL).	High accuracy with low computational cost, suitable for mobile devices.
FakeCatcher: **Commercial**	Intel	Uses physiological signals (e.g., heart rate) for deepfake detection.	High precision in real- world scenarios, nonintrusive.
DeepSight: **Commercial**	Fraunhofer Heinrich Hertz Institute	Uses multi-modal data, integrates visual and auditory features.	High robustness across various deepfake generation techniques.
TruthGuard: **Commercial**	NVIDIA	AI-based real-time deepfake detection, leverages GPU acceleration.	High performance and speed, designed for high throughput environments.

Table 7, Table 8, Table 9, Table 10, Table 11, Table 12, Table 13 provide a comprehensive overview of the various strategies and techniques utilized by different authors in addressing the challenge of detecting fake information within datasets. The techniques discussed in this study encompass several domains such as computer vision, deep learning, and audio-visual analysis. Each author in this research offers a distinct methodology to tackle the difficulties associated with manipulated material. For example, certain researchers prioritize the task of distinguishing fabricated information from authentic data, while others investigate discrepancies at both the global and local levels within deepfake datasets. There are various methodologies that aim to tackle issues related to disparities between audio and visual stimuli, manipulations involving multiple sensory modalities, and faults in spatial and temporal perception. Moreover, the incorporation of techniques such as attention mechanisms [42], feature extraction [43], and self-supervised learning assumes significant importance in the identification of forgeries. These methods are complemented by the utilization of dedicated forgery feature extractors and motion magnification techniques.

**Table 7:** A Comprehensive Review of Deepfake Detection Methods and Performance Evaluation.

Sr.	Methodology/Techniques	Dealing With	Forgeries Identified	Dataset	Performance Parameter
[44]	ADAL - Disentanglement Generator, ACCL, Discriminators and Single Scale Feature Separator	Videos/Images	Interference of irrelevant information and artifacts in the fake faces	FF++, DFD, DFDC and Celeb-DFv2	AUC, ACC
[45]	AMSIM - Global Inconsistency and a View (GIV) more meticulous Multi-timescale Local Inconsistency View (MLIV)	Videos	Indetectable local spatiotemporal abnormality	FF++, DFDC, DFD Celeb-DFv2	AUC, ACC
				DF1.0-Raw DF1.0-Per and Wilddeepfake	
[46]	Spatial-temporal model - Long-distance attention (Spatial Attention Module and Temporal Attention Module)	Videos	Global semantic inconsistency Spatial and temporal defects,	Celeb-DF FF++ and	AUC, ACC
[47]	AdapGRnet - Manipulation trace extractor (MTE), Attention fusion mechanism(AFM)	Images	Manipulation Traces, Face Forgeries ,Low-quality visual content	HFF, FF++, Celeb-DF and DFDC	AUC, ACC
[48]	Transformer-based self supervised learning	Videos/	Masked patches, FaceShifter, Deepfakes	FF++,	AUC, EER
	(Intra-Consistency and Inter-Diversity) a: Self Prediction Learning (SPL), Adjustable Forgery Synthesizer (AFS)	Images		Celeb-DF DFDC and UADFV	
[49]	Dynamic fine-grained difference capture module(DFDC-module) and a multi-scale spatio temporal aggregation module (MSA-module) spatio– temporal denoising operation:correlation,fine- grained	Videos	Spatio-temporal inconsistency.	FF++, Celeb-DF and DFDC	AUC, ACC F1-Score
[50]	Motion magnification, 3D Residual-in-Dense ConvNet: Compression, Downsampling, Average Pooling	Videos	Heavy compression, face-swapping, Highlighted Artifacts	FF++, Celeb-DF	AUC, EER Precision

**Table 8:** A Comprehensive Review of Deepfake Detection Methods and Performance Evaluation (Continue).

Sr.	Methodology/Techniques	Dealing With	Forgeries Identified	Dataset	Performance Parameter
[51]	DFDM(Deepfake Detection Model)-DCGAN architecture: Bleach Generator, CrossEntropy Loss	Videos/ Images	Bleached/Compressed Images	FF++,	AUC, ACC
[52]	Transferable Cycle Adversary Generative Adversarial Network (TCA GAN)(reconstruction autoencoder) post- regularization module, Adversial perturbation	Videos/ Images	Face-Swapping	CelebA and Face-Scurb	BRISQUE, Accuracy
[53]	FCAN-DCT: Compact Feature Extraction (CFE)module and Frequency Temporal Attention (FTA) module.	Videos	Spectrum spatial temporal frequency clue.	FF++, CelebA, Wild Deepfake Own Dataset – DeepfakeNIR	AUC, ACC
[54]	Multi-CNN: Resnet50, Densenet121 and Inception ResnetV2, Convolutional Block Attention Mechanism (CBAM)	Videos/ Images	Forgery cues	DFDC	Accuracy, Precision and F1 Score
[55]	BTS-E: TTS, Sound Seg- mentation Phase, Synthetic Speech Detection Phase	Audio	Deepfake speech	ASVspoof2019	EER and min-tDCF
[56]	AVFakeNet DST-Net (Input Block, Feature Extraction Block, Output Block)	Audios/ Videos	Manipulation in audio and visual streams	FakeAVCeleb and Celeb DF	EER and min-tDCF
[57]	AVoiD-DF Temporal Spatial Encoder (TSE), a Multi-Modal Joint Decoder(MMD), and a Cross-Modal Classifier.	Video	Audio-visual inconsistency	DefakeAVMiT, FakeAVCeleb and DFDC	ACC, AUC
[58]	CNN, Generalization Deepfake Detector (GDD), Soft-pair, Classification Loss (SCL), alignment loss (CAL)	Video/ Images	Highlighted Artifacts	CelebDF, DFDC and FF++	ACC, AUC

**Table 9:** A Comprehensive Review of Deepfake Detection Methods and Performance Evaluation (Continue).

Sr.	Methodology/Techniques	Dealing With	Forgeries Identified	Dataset	Performance Parameter
[59]	MobiDeep-Training Data Annotation (TDA), CornealSpeculart Backscatter Detection(CSBD), and Feature Extraction and Classification (FEC)	Images	Facial Image Environmental Parameters	MobiDeep- DFD,	Accuracy, Loss
[60]	DMA-STA - Feature extraction from multiple single frames based on SAM(Spatial Attention Map), Video-level fusion module based on TAM (Temporal Attention Map)	Videos	Spatio-temporal inconsistency.	DFDM	Accuracy
[61]	SWYNT - SVM, HOG, encoder block, bottleneck block, decoder block,and skip connections having Swin Transformer.	Videos	Identity swap and puppet mastery.	FF++ and Celeb-DF	Accuracy, AUC
[62]	Generative adversarial networks (GANs), LeakyReLU	Images/Videos	Facial reenactment	DDFD, Deepfake TIMIT, CASIA- WebFace and FFHQ	Accuracy
[63]	Non-negative constrained classifier (NCC), Multiclass forgery-domain classification, Augmentation integration module (AIM)	Videos	Discriminative forgery relevant information: Augmented Faces	DDFD, FF++ and Celeb-DF	AUC
[64]	Dual Attention Forgery Detection Network (DAFDN) - Spatial reduction attention block (SRAB), Forgery feature attention module (FFAM)(AIM)	Videos	Global Inconsistency, Illumination Estimation, Geometry Estimation, Warping traces.	FF++ and DFDC	AUC
[65]	Meta-learning-MDD: metaweight learning and optimization, pair-attention loss(PAL) and average-center alignment loss(ACA).	Videos/ Image	FaceSwap and Neural- Textures.	FF++, DFDC and Celeb-DFv2	AUC, ACC and loss
[66]	Specific Forgery Feature Extractors (SFFExtractors), U-net structure(triplet loss, location loss, classification loss, and automatic weighted loss) and Common Forgery Feature Extractor (CFFExtractor).	Videos	Face boundary warp, Noise, FaceSwap and Neural Textures	FF++, DFDC and CelebDF	AUC, ACC

**Table 10:** A Comprehensive Review of Deepfake Detection Methods and Performance Evaluation (Continue).

Sr.	Methodology/Techniques	Dealing With	Forgeries Identified	Dataset	Performance Parameter
[67]	Transformer-based framework with feature compensation and aggregation (Trans-FCA): Locality Compensation Block (LCB), Global-Local Cross-Attention (GLCA), Multi-head Clustering Projection (MCP) and Frequency- guided Fusion Module (FFM)	Videos/ Images	Forgery cues	FF++ and CelebDF	AUC, ACC
[68]	Mel-frequency cepstral coefficients (MFCCs), SVM, VGG-16	Audio	Synthetic audio	Fake-or- Real and	Accuracy
[69]	Speaker verification - Centroid-based Testing(Speaker Embedding Extractor, Centroid Computation), Multisimilarity Testing	Audio	Synthetic and spoofed audios	ASVSpoof2019 FakeAVCelebV In-The- Wild Audio Deepfake dataset	, Accuracy 2,
[70]	CNN - ReLu, MFCC, STFT, FFT, Spectrogram parameterization.	Audio	Synthetic speech	ASVSpoof2017	, ROC, TPR, FPR
[71]	Extracting face edge bands(Convex Hull, Dilation, Erosion, Bitwise Not Algorithm), EfficientNet-B3	Videos	Forged videos	FF++	AUC
[72]	Quantum-Inspired Evolutionary, AlexNet	Images	Manually created fake- face	Own Dataset	Accuracy
[73]	CNN, GAN, Confu- sion Matrix(provided. Resnet, Resnext50 and LSTM)	Videos	Visual Artifacts	FF++ and DFDC	Accuracy
[74]	SRM Filter Layer, NA-VGG	Images	Image Augmentation	CelebDF, FF++ and UADFV	Accuracy
[75]	MTCNN, Data Augmentation, EfficientNet- b0Resnext50 and LSTM)	Videos	Forged videos	FF++	AUC

**Table 11:** A Comprehensive Review of Deepfake Detection Methods and Performance Evaluation (Continue).

Sr.	Methodology/Techniques	Dealing With	Forgeries Identified	Dataset	Performance Parameter
[76]	Speech Emotion Recognition(SER), Synthetic Speech Detector(SSD)	Audios	Audio Spoofing	ASVspoof 2019, LibriSpeech, LJSpeech, Cloud2019 and IEMOCAP	ROC, TPR, FPR
[77]	BA-TFD: Video Encoder - 3DCNN, Audio Encoder - 2DCNN, Contrastive and Cross Entropy. Loss	Audios/ Videos	Temporal forgery localization	BMN, AGT, AVFusion, MDS and DFDC	AUC
[78]	Spatiotemporal Convolutional Network (SCN), Photo-Response Non- Uniformity (PRNU) analysis	Videos	Forged videos	FF++, CelebDF and FaceHQ	Accuracy
[79]	CNN - DCT, Xception, softmax function	Images	Highlighted Artifacts	OpenForensics	Accuracy
[80]	SpecRNet- residual block (ResBlock) and FMS attention block	Audio	Artificially modified audio	LJSpeech and Japanese JUST	AUC, EER
[81]	FD-DBN and FD-DG: CSSM, Global and Local Feature Extractor	Videos	Temporal forgery localization	FF++, Celeb-DF- v2, DFDC, DFD and Dfo	AUC, ACC
[82]	MaskGAN - U-Net, SSE, DeeplabV3+	Images	Face Swapping	FF++, CelebA and CelebDF	AUC
[83]	CNN-MFCC, Mel-spectrum, Chromagram, and spectrogram, generative adversarial networks (GAN)	Audio	Deepfake speech	VCTK and LibriSpeech	Accuracy
[84]	Generative adversarial networks(GAN)-GAN discriminator, ReLU, MTCNN	Videos	Forged videos	Deepfake	Accuracy
[85]	XLS-R, ECAPA-TDNN	Audio	fake audios.	Own Dataset	EER

**Table 12:** A Comprehensive Review of Deepfake Detection Methods and Performance Evaluation (Continue).

Sr.	Methodology/Techniques	Dealing With	Forgeries Identified	Dataset	Performance Parameter
[86]	CNN - dlib, Inception- ResNetV2, MobileNet, DenseNet121, softmax	Videos	Short and Low Resolution Deepfake Video	FF++ and DFDC	Accuracy, Precison and Recall
[87]	ResNext Convolution Neural Network, LSTM, Correlation	Videos	Synthetic content	DFDC	Accuracy
[88]	WaveletCNN and VGG16 - additive margin softmax loss (AM Softmax)	Audio	Audio spoofing	ASVspoof2019 and ASVspoof2021	EER, tDCF
[89]	Preprocessing Stage: Mel- Frequency Cepstral Coefficients (MFCC), (Speech Denoising)DNN: CNN, Multi-Layer Perceptron's (MLPs), STFT	Audio	DSynthetic speech	Urban- Sound8K, Conversational AMI- Corpus, and FakeOr- Real+	Accuracy ,
[90]	DeepfakeNet, VGG19	Videos/Images	FaceSwap	FF++, TIMIT and Kaggle	ROC, AUC
[91]	Dlib Face Extractor, XceptionNet, Bidirectional LSTMs - Cross entropy, KL Divergence.	Audios/ Videos	Spatial and temporal signatures	Video:FF++, Celeb-DF, Audio: ASVSpoof	t-DCF, EER
[92]	DeepfakeStack, GAN	Images	Manipulation Traces, Face Forgeries	FF++	Accuracy
[93]	Large margin cosine loss function (LMCL), online frequency masking augmentation, ResNet	Audio	Spoofed audio	ASVspoof 2019	t-DCF, EER
[94]	CNN CT Extraction: E-step(Update Variable), M-step(Update Hypothesis), Random Forest	Images	Convolutional Traces	CELEBA, FF++, IMLE and SPADE	Accuracy Average Precision

**Table 13:** A Comprehensive Review of Deepfake Detection Methods and Performance Evaluation (Continue).

Sr.	Methodology/Techniques	Dealing With	Forgeries Identified	Dataset	Performance Parameter
[95]	FCD-Net: facial synaptic saliency module (FSS), contour detail feature extraction module (CDFE), and the distinguishing feature fusion module (DFF)	Images	homologous deepfake face images	HDFD	Accuracy
[96]	Frequency spectrum and context color channels analysis	Images	spectral anomalies and statistical features	FF++, Celeb-DF	Accuracy
[97]	CNN, RNN, Image preprocessing, Matlab Stimulator	Images	Forged videos	Deepfake dataset	Accuracy

**Fig. 6:**
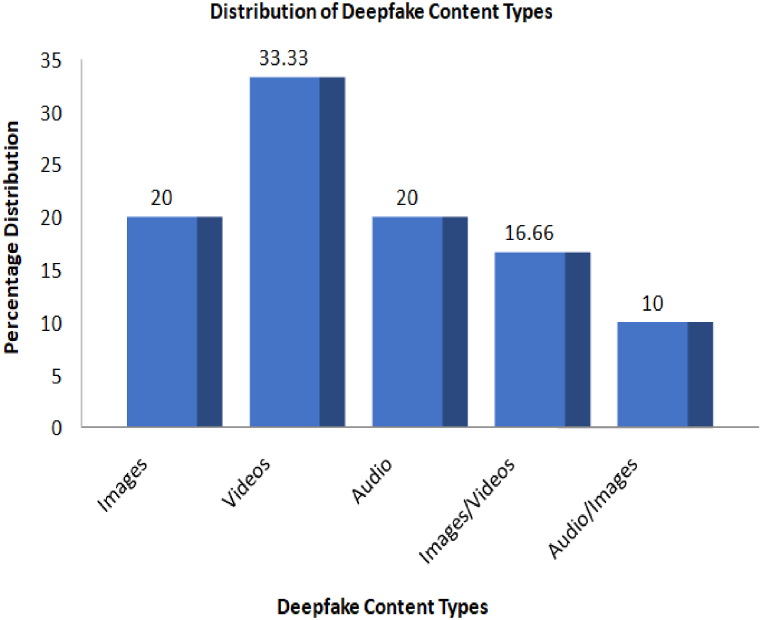
Distribution of deepfake Content Types based on the Reviewed Studies.

Fig. 6, presents a breakdown of content types within papers we have surveyed, crucial for surveying and researching deepfake detection. The findings of our analysis indicate that a significant proportion of the papers, specifically 20 %, have a primary emphasis on images. In contrast, the majority of the papers, around 33.33 %, predominantly concentrate on videos. These results underscore the prominence of dynamic media in the realm of deepfake identification research. Moreover, it is worth noting that a considerable proportion, specifically 20 %, of the reviewed papers focus on audio-related factors, thus highlighting the critical role of audio components in the analysis of deepfakes. It is worth noting that a significant proportion of the publications, specifically.

16.66 %, examine the subject matter of images as well as videos. This observation highlights the multifaceted characteristics of deepfake material that have been extensively explored in the existing body of literature. Ultimately, a notable proportion of the examined scholarly articles, specifically 10 %, delve into the convergence of both visual and audio elements, exemplifying the wide range of methodologies employed in addressing the multifaceted obstacles associated with multimodal deepfake phenomena. The analysis presented here enhances our survey by providing insights into the many content modalities that have been examined in the reviewed literature.

**Fig. 7:**
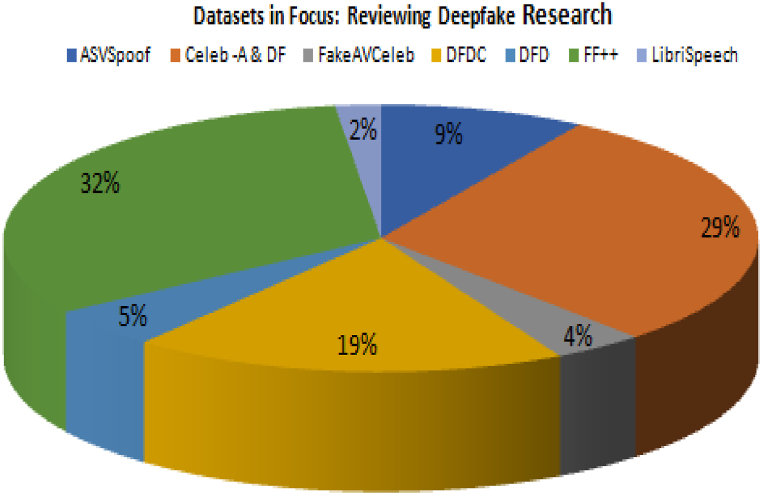
Distribution of Datasets used for deepfake Research based on the Reviewed Studies.

Fig. 7, provides a comprehensive overview of review of diverse datasets in the realm of deepfake research, offering valuable insights. The provided analysis provides insight into the distribution patterns of these datasets within the research papers that have been examined, indicating their significance and effectiveness in furthering our comprehension of deepfake technology. The presence of ASVSpoof [98] was detected in 9 % of the papers, suggesting its involvement in targeted research investigations. Significantly, Celeb-A and DF [99] emerged as prominent entities in our dataset evaluations, being referenced in almost 30 % of the scholarly literature. This underscores the extensive acceptance and importance of deepfake technology within the scholarly community. The dataset known as FakeAVCeleb [99] was mentioned in a relatively small proportion of the papers, specifically 4 %. The DeepFake Detection Challenge (DFDC) [99], a significant asset for identifying deepfake content, garnered attention in around 19 % of academic papers, underscoring its pivotal importance in facilitating research progress. The significance of DFD [100], albeit relatively diminished, was nevertheless observed in approximately 5 % of the papers. The FF++ [100] dataset has been identified as the most often examined dataset, appearing in 33 % of the academic papers. This highlights its extensive coverage and widespread adoption in the analysis of deepfake technology. Finally, it is worth noting that LibriSpeech [100], although it is not as commonly referenced, was identified in 2 % of the research publications, suggesting that it may have some significance in the field of audio analysis. In conclusion, the dataset evaluations conducted highlight the wide range of sources that have been examined in deepfake research.

**Fig. 8:**
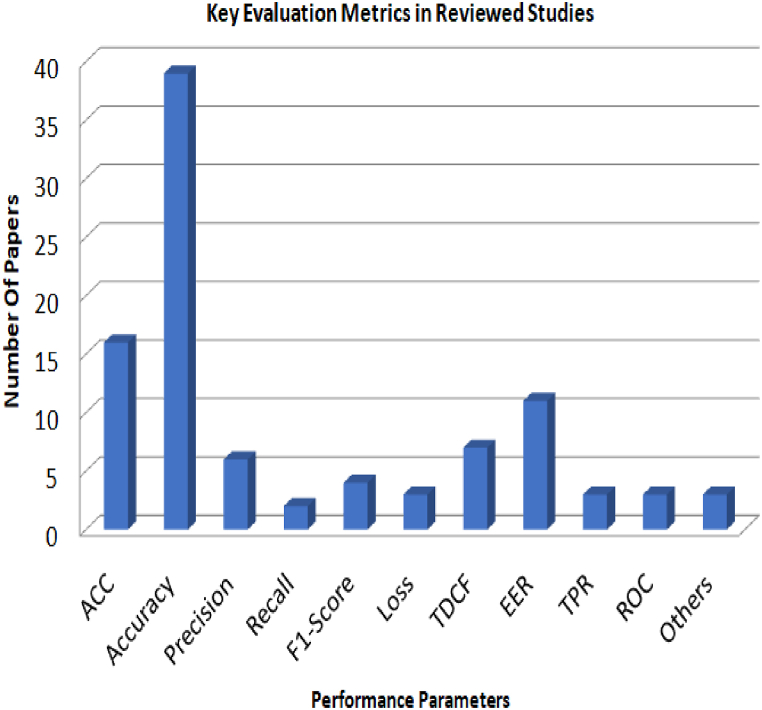
Distribution of Key Evaluation Metrics in deepfake Research based on the Reviewed Studies.

Fig. 8, provides a detailed overview of the various evaluation metrics used to assess research papers, most likely within the realms of machine learning and data analysis. These metrics are crucial performance and efficacy indicators for predictive models. Notably, the Area Under the Curve (AUC) [101] metric has a significant value of 27, highlighting its utmost significance in model evaluation. In addition, ACC [101] and a repeated measure of Accuracy [101] with values of 16 and 39, respectively, emphasize the importance of precise classification in the reviewed research papers. Precision, Recall, and F1-Score metrics [102], with respective values of 6, 2, and 4, illuminate the nuanced analysis of true positive and false positive trade-offs in model evaluations. Typically, a loss value of 3 represents the error in model predictions. In addition, it contains TDCF, EER, TPR, and ROC metrics [102], indicating an emphasis on timedependent or classification tasks. The others indicate the metrics that were used as a evaluation metrics but in a negligible terms, with a repeated value of 3, indicates that additional metrics or factors are being considered within the context of the research. Overall, this Fig. 8 demonstrates a comprehensive and multifaceted evaluation of model performance, highlighting the meticulous analysis of various aspects of model precision and efficacy in the reviewed research papers.

## 6 Case study

In 2023, a significant deepfake fraud incident occurred in Hong Kong, where scammers used deepfake technology during a video conference to impersonate a company's Chief Financial Officer (CFO). The impersonation was so convincing that a finance worker in the company was deceived into transferring $39 million to the fraudsters. This case highlights the dangers of deepfake technology being used for large-scale financial crimes and how sophisticated these attacks have become.

**Location:** Hong Kong.

**Industry:** Finance/Corporate Sector.

**How the Attack Happened**: The scammers utilized deepfake video and audio technology to create a realistic digital version of the CFO, replicating both their appearance and voice. During a virtual meeting, the scammers convinced the finance worker that the transfer was a legitimate request from the CFO. The worker, trusting the authenticity of the deepfake, initiated the transaction of $39 million to the fraudsters’ accounts.

Impact.

• Financial Loss: The company suffered a direct loss of $39 million. Given the difficulty in tracking and recovering funds in cases of cybercrime, this loss is likely irrecoverable.
• Corporate Trust Issues: The use of deepfake technology in a corporate environment eroded trust in virtual communications, especially in industries where remote work and digital interactions are prevalent.
• Broader Implications for Cybersecurity: This incident alarmed companies worldwide about the risks of deepfake technology in financial transactions. It highlighted the need for enhanced verification methods beyond video conferencing and traditional communications, as these can no longer be trusted without multi-factor authentication.
• Legal Repercussions: Though no specific legal details were made public, incidents like these typically trigger investigations by local law enforcement and possibly international agencies, given the cross-border nature of the crime.

Key Takeaways.

• Sophistication of Deepfake Technology: The deepfake video was so realistic that it bypassed the standard trust mechanisms employees rely on. This shows the advancing quality of deepfakes and their potential for harm.
• Corporate Vulnerabilities: Companies relying on digital communications, especially for financial transactions, are at high risk of such attacks.
• Need for Advanced Authentication Protocols: This case emphasized the importance of introducing multi-factor authentication (MFA) or other advanced verification processes to prevent fraud, especially when large sums of money are involved.

**Response and Preventive Measures:** Following the incident, security experts suggested that businesses adopt more robust verification processes for sensitive operations, including biometric authentication or encrypted digital signatures. Governments and companies are also becoming more aware of the need to update cybercrime laws to include regulations around deepfake technology.

**Global Awareness**: This case not only had local consequences but also raised global awareness about the increasing use of deepfakes in corporate fraud. Financial institutions and corporations worldwide began reviewing their cybersecurity policies and procedures for verifying high-value transactions in digital environments. This Hong Kong case is a prime example of how advanced cybercriminals are becoming with deepfakes, pushing organizations to rethink security in the age of digital and virtual workspaces.

## 7 Applications of deepfake

**Fig. 9:**
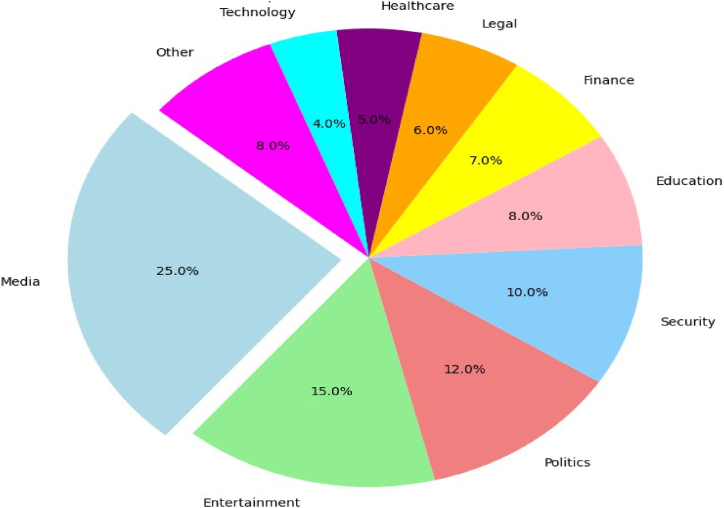
Use cases of deepfake across industries.

Deepfake technology, despite being commonly associated with malicious applications and disinformation, has a variety of potential applications across industries. Deepfake technology has applications in the entertainment, marketing, education, and healthcare industries. It can generate Computer-Generated Imagery (CGI) characters, generate realistic special effects for movies and television, and engage audiences in brand advertisements. It can also be used for language acquisition, allowing users to converse with characters who speak various languages. Fig. 9 provides the use cases of deepfake across industries highlighting the applications of deepfake.

Deepfake simulations can replicate surgeries, procedures, and patient interactions within the healthcare industry, ensuring the safety of medical students and professionals [103]. Language learners can utilize deepfake characters for practice. Sign language interpreters, voice assistants such as Siri and Alexa [103], and localized pronunciation improve accessibility. Brands can use social media to sell their goods and services by making “virtual influencers”. Re constructing the past can be accomplished through educational experiences, museums, and virtual excursions. Using deepfakes, artists can create surreal artworks, videos, and performances. While maintaining lip synchronization accuracy, dubbing and localization can make films, television programs, and video games accessible internationally.

Deepfake technology can aid in the preservation of cultural heritage by restoring and enhancing damaged audiovisual content. For special occasions like birthdays, marriages, and anniversaries, personalized content can be created. By incorporating realistic facial expressions and natural language processing, chatbots and virtual avatars can enhance customer service. Deepfakes can be used in research to generate synthetic data for experiments, simulations, and studies with limited or restricted access to actual data.

However, it is essential to recognize the potential negative applications and hazards of deepfakes. Deepfakes can be used to create fake news, manipulate information, and pose a threat to public trust. Privacy and consent concerns arise as realistic fake content can lead to harm, harassment, or exploitation without an individual's consent. They can also enable impersonation, leading to online scams, social engineering attacks, and political manipulation. Fraudsters can deceive individuals or organizations, causing financial loss or reputational damage. The ease of creating convincing fake content poses a risk to individuals' or organizations' reputations, as false information or damaging content can be easily shared, causing significant harm.

## 8 Ethical implications of deepfake

**Fig. 10:**
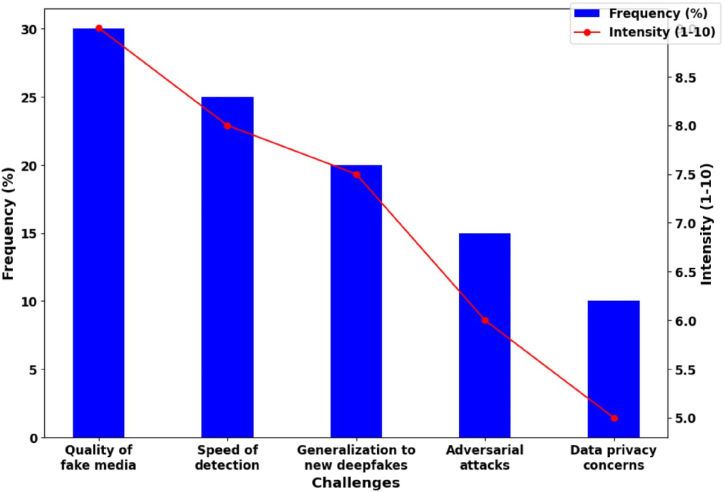
Frequency and intensity of challenges in detecting deepfakes.

As a result of advancements in artificial intelligence and machine learning, deepfake technology is a potent instrument in the digital realm. Fig. 10 shows the frequency and intensity of challenges in detecting deepfakes that drives as a reason causing ethical implications. It involves altering digital content intentionally to create synthetic depictions of individuals or events, which can lead to deception, the dissemination of false information, and societal damage [104]. The ethical implications of deepfake technology include violations of privacy and consent, erosion of media credibility, and manipulation of political discourse. Deepfakes are particularly significant in news and social media, where false information can propagate rapidly. Deepfakes frequently involve the unauthorized use of individuals' likenesses and accents, thereby constituting a widespread invasion of privacy. A person's reputation and livelihood can be harmed by the creation of fabricated videos or audio recordings depicting them engaging in illegal or demeaning behavior.

Deepfakes pose a threat to the democratic process and the integrity of political systems by influencing political discourse through the creation of fake speeches or interviews featuring prominent political figures. Ethical considerations regarding consent and consent forgeries center on permission and the degree to which individuals can exercise control over their digital identities. Deepfakes have the potential to undermine public faith in journalism, leading

to increased skepticism and societal fragmentation. In criminal cases, they can be used to fabricate alibi or tamper with evidence, confronting the pursuit of truth by law enforcement agencies. Due to the rapid development of deepfake technology, it is necessary to strike a balance between safeguarding against misuse and upholding the principles of free speech. The creation of effective laws and regulations is essential, but presents considerable obstacles.

## 9 Conclusion and future work

The rapid development of deepfake technology has ushered in an era of heightened concerns over the veracity of digital media content. The proliferation of deepfakes, which can seamlessly blur the boundaries between reality and deception, poses significant threats across multiple domains, such as the media, politics, and personal safety. This comprehensive study investigates the development and evaluation of autonomous methods for detecting and evaluating deepfake media. Our investigation has focused on cutting-edge techniques and tools for image, video, and audio-based content. We have thoroughly evaluated the effectiveness of the underlying technologies, including deep learning models, in distinguishing between authentic and manipulated media. In addition, our investigation has led us to investigate emerging detection strategies, utilizing sophisticated machine learning, computer vision, and audio analysis. In an era where the ability to distinguish between fact and fiction is of the utmost importance, our mission is to contribute to a more secure and knowledgeable digital ecosystem by advancing the understanding and application of autonomous detection and evaluation methods.

To address on future directions, improving detection accuracy with small datasets is a crucial future goal that may be tackled with the use of data augmentation, transfer learning, and self-supervised learning methods. Additionally, it is critical to construct lightweight models capable of real-time analysis on edge devices such as smartphones, as real-time deepfake detection becomes vital for live video or streaming applications. Improving cross-modal detection which detects advanced deepfakes by analyzing discrepancies in text, video, and audio is another crucial direction. Since deepfake approaches are evolving quickly, research must also concentrate on developing models that are resistant to adversarial attacks, maybe through adversarial training methods. A major research gap in lack of standardized datasets representing real-world deepfake scenarios across multiple platforms and qualities especially low-resolution or compressed media is identified. In addition, even though detection models have produced encouraging results, there is a gap in explainability because many of them rely on ”black box” deep learning techniques, which restrict how results may be interpreted. One problem associated with the rapid advancement of GAN-based approaches is the requirement for detection models to improve their generalization across various GAN designs without requiring regular retraining. In order to ensure the ethical deployment of deepfake detection systems while addressing privacy concerns, it is necessary to match technological breakthroughs with legislative frameworks. These domains underscore the necessity of additional investigation to push the field of deepfake detection forward.

In the future, we will focus primarily on enhancing the efficiency of deepfake detection through the integration and fusion of multiple techniques. We intend to accomplish this by combining conventional and sophisticated deep learning techniques to produce a more robust and accurate detection model. Combining distinct classification and deepfake detection algorithms will be required. A major emphasis will also be placed on developing a detection model that is transparent, meaning that it can explain how it makes judgments. This strategy seeks to enhance the overall dependability and credibility of deepfake detection systems.

## CRediT authorship contribution statement

**Reshma Sunil:** Writing – original draft, Methodology, Conceptualization. **Parita Mer:** Writing – original draft, Methodology, Conceptualization. **Anjali Diwan:** Writing – original draft, Methodology, Formal analysis, Conceptualization. **Rajesh Mahadeva:** Writing – original draft, Supervision, Methodology, Conceptualization. **Anuj Sharma:** Writing – original draft, Supervision, Methodology, Conceptualization.

## Data availability statement

No new data was generated for the research described in the article.

## Declaration of competing interest

The authors declare the following financial interests/personal relationships which may be considered as potential competing interests:Author: Anuj Sharma is noted as an AE of this journal.
